# Carbon Dots and Their Films with Narrow Full Width at Half Maximum Orange Emission

**DOI:** 10.3390/molecules29204787

**Published:** 2024-10-10

**Authors:** Jiangchen Wu, Jianan Liu, Xinghua Liu, Jingxia Zheng, Lin Chen, Yongzhen Yang, Chunhui Su

**Affiliations:** 1 School of Material Science and Engineering, Changchun University of Science and Technology, Changchun 130022, China; wujiangchen0219@126.com; 2 Key Laboratory of Interface Science and Engineering in Advanced Materials, Ministry of Education, Taiyuan University of Technology, Taiyuan 030024, China

**Keywords:** phloroglucinol, orange-emissive carbon dots, fluorescent films, narrow full width at half maximum

## Abstract

To obtain carbon dots (CDs) with narrow full width at half maximum (FWHM) and long-wavelength emission, carbon sources with high conjugate sizes and abundant functional groups can be employed to synthesize CDs. In this study, orange-emissive carbon dots (OCDs) were synthesized with phloroglucinol and rhodamine B as precursors. When the molar ratio of them was 30:1, and ethanol was served as the solvent, OCDs with optimized emission wavelength at approximately 580 nm, an FWHM of 30 nm, and a quantum yield (QY) of 27.31% were obtained. Subsequently, the OCDs were incorporated into polyvinyl alcohol (PVA) to fabricate solid-state OCD/PVA fluorescent films, which exhibited an FWHM of 47 nm. The PVA matrix facilitated the dispersion of OCDs, thereby suppressing non-radiative energy transfer among the OCDs and enhancing luminescence efficiency. Consequently, compared with OCDs, the OCD/PVA film exhibited significant luminescent enhancement, and the QY of the composite film was increased to 84.74%. Moreover, OCD/PVA film showed good transmittance and thermal stability. This research offers a solid theoretical and experimental foundation for the potential applications of CDs in the field of solid-state lighting.

## 1. Introduction

Carbon dots (CDs) are a novel type of carbon nanomaterials with a size of less than 10 nm and generally exhibit a spherical or spherical-like morphology, which is composed of a carbonized core and diverse surface state with various functional groups. Owing to the advantages of high stability, low cost, low toxicity, and exceptional and adjustable luminescence performance, CDs have shown great potential in various fields such as chemical sensing, bio-imaging, solar cells, and optoelectronic devices [1,2]. However, most CDs exhibit fluorescence quenching in solid-state because of the aggregation-caused quenching (ACQ) effect, which severely limits their application in the field of solid-state lighting, such as lasers and light-emitting diodes (LEDs) [3,4]. Currently, researchers have indirectly achieved the solid-state luminescence of CDs through methods such as matrix dispersion and surface engineering. Alternatively, the direct preparation of aggregation-induced luminescent CDs, as well as in situ dispersion and in situ surface engineering, has been employed to obtain solid-state luminescent CDs. The majority of current solid-state luminescent CDs exhibit a wide full width at half maximum (FWHM). Actually, fluorescent materials with FWHM smaller than 60 nm show high color purity when they are used for lasers and electroluminescence devices. Therefore, developing solid-state luminescent CDs with narrow FWHM will demonstrate great potential in the application of lasers and LED devices [3,5,6,7].

Narrow FWHM solid-state emissive CDs can serve as a gain medium for lasers, enhancing their luminescence efficiency and, as a luminescent layer, improving the color purity of electroluminescent LEDs [7,8]. Therefore, the synthesis of narrow FWHM, solid-state, emissive CDs with high luminescence performance holds significant importance in enhancing device performance. Current studies on narrow FWHM emissive CDs primarily concentrate on the solution state [7,9]. However, there have been related reports on narrow FWHM solid-state emissive CDs. Yoshinaga et al. [10] prepared CDs by heating phloroglucinol in a glycol solution of 1,2-pentanediol and, then, dispersed them in polyvinyl alcohol (PVA) and polyvinyl pyrrolidone (PVP) matrices to achieve solid-state luminescence, and blue fluorescence (474 nm) and green fluorescence (494 nm) under UV excitation with FWHM values of 33 and 35 nm were obtained, respectively. Yuan et al. [11] used resorcinol as a raw material to synthesize green-emissive CDs (520 nm) and red-emissive CDs (610 nm) with FWHM values of 31 and 33 nm by adjusting the reaction time, respectively. Subsequently, solid-state luminescence was achieved by embedding green- and red-emissive CDs into poly (methyl methacrylate) matrices, and their fluorescence emission and FWHM values did not change significantly compared with those in the solution state. However, these reports did not investigate the fluorescence quantum yield (QY) of solid-state luminescent CDs. Xu et al. [12] achieved fluorescence emission in the UV-B region (308 nm) by employing acetic acid to promote the generation of sp^3^ bonds and reducing the size of the sp^2^-conjugated structure during the synthesis of CDs. Consequently, the sp^2^ domains were effectively separated by the sp^3^ domains, reducing the ACQ effect. As a result, the solid-state luminescence of CDs was achieved, and the QY of CDs reached 20.2% with an FWHM of 24 nm. However, the emission wavelengths of narrow FWHM solid-state emissive CDs are primarily concentrated in the short-wavelength region (≤520 nm), which hampers their application in the field of solid-state luminescence. Moreover, the fluorescence QY narrow FWHM solid-state emissive CDs still need to be improved, which will impede further enhancements in the efficiency of solid-state lighting. Therefore, developing long-wavelength solid-state CDs with both high QY and narrow FWHM holds paramount importance for achieving high-efficiency solid-state lighting devices.

Currently, small-molecule carbon sources containing benzene rings play a significant role in producing CDs with narrow FWHM and high fluorescence QY, owing to their sp^2^-conjugated structure [13]. Moreover, some dye-molecule-derived CDs may inherit the optical properties of the dye, thus improving the optical properties of the CDs [14]. Phloroglucinol has a benzene ring structure and abundant hydroxyl functional groups, which makes it beneficial for preparing red-/orange-emissive CDs [15]. Meanwhile, rhodamine B exhibits a narrow FWHM and a long-wavelength emissive fluorescence and has improved the fluorescence property of as-prepared CDs as a functional modifier [14], which can endow CDs with excellent fluorescence characteristics of both a narrow FWHM and a long-wavelength emission. Therefore, this study proposed that phloroglucinol and rhodamine B were together used as precursors to synthesize the orange-emissive CD (OCD) solution with a narrow FWHM of 30 nm by a convenient and rapid microwave method, accompanied by property optimization by adjusting reactant molar ratio and solvent type. Subsequently, with the aim of realizing highly efficient, solid-state, emissive CDs with narrow FWHM, a OCD/PVA film with a QY of 84.74% and an FWHM of 47 nm was obtained. This work will provide a new idea for the application of OCDs in solid-state luminescent devices.

## 2. Results and Discussion

### 2.1. Optimized OCDs with Narrow FWHM

**Figure 1:**
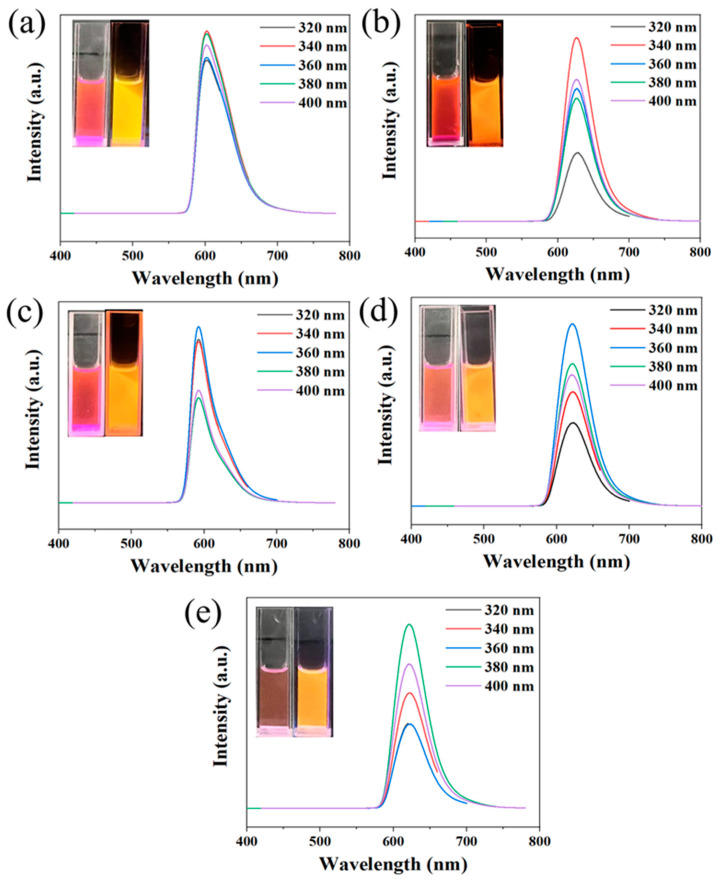
PL spectra of OCDs (0.3 mg/mL) synthesized with phloroglucinol and rhodamine B at different molar ratios (**a**) 75:1, (**b**) 30:1, (**c**) 7.5:1, (**d**) 1.5:1, and (**e**) 0.75:1 using water as the solvent (inset photos of OCDs aqueous solution under daylight (left) and ultraviolet lamp (right)).

To obtain narrow FWHM CDs, the effects of different reactant molar ratios and different solvents (water and ethanol) on the FWHM of CDs were investigated. First, the reaction solvent of water was selected, and the molar ratio of phloroglucinol and rhodamine B were regulated by 75:1, 30:1, 7.5:1, 1.5:1, and 0.75:1 to synthesize narrow FWHM CDs. Their photoluminescence (PL) spectra were measured to investigate the effect of the molar ratio of precursors on the FWHM of the CD emission (as shown in Figure 1). It demonstrates that all CDs exhibit excitation-independent properties at an emission peak of about 600 nm, which belongs to orange emission. The FWHM are 48 (Figure 1a), 35 (Figure 1b), 36 (Figure 1c), 46 (Figure 1d), and 51 nm (Figure 1e), respectively, which are all less than 60 nm. When the molar ratio of phloroglucinol and rhodamine B is 30:1, the obtained OCDs have the narrowest FWHM (35 nm).

**Figure 2:**
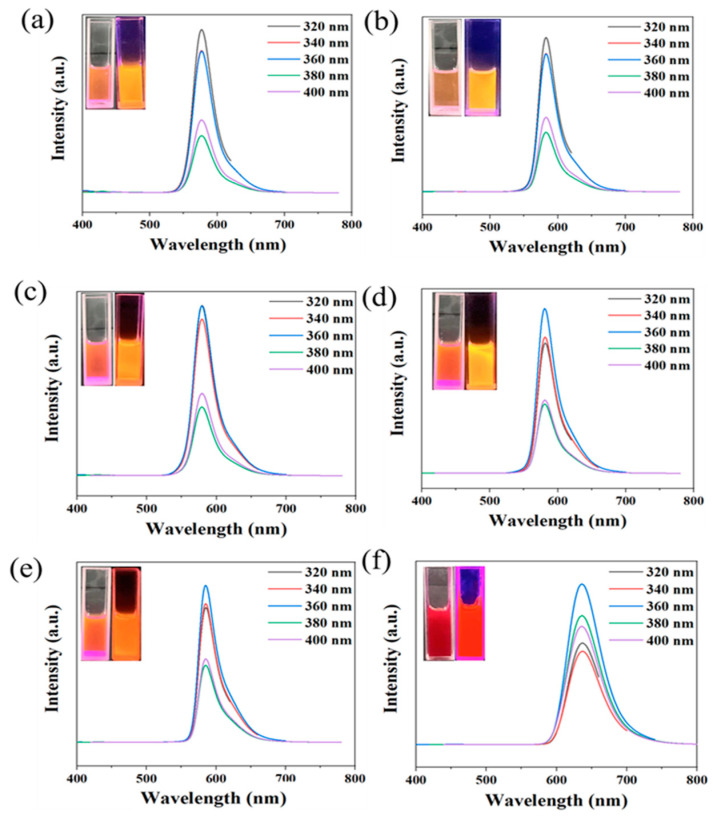
PL spectra of OCDs (0.3 mg/mL) synthesized with phloroglucinol and rhodamine B at different molar ratios (**a**) 75:1, (**b**) 30:1, (**c**) 7.5:1, (**d**) 1.5:1, and (**e**) 0.75:1 using ethanol as the solvent; (**f**) PL spectra of rhodamine B in ethanol (inset photos of OCDs ethanol solution under daylight (left) and ultraviolet lamp (right)).

Subsequently, the reaction solvent was changed to ethanol to synthesize CDs at reactant molar ratios of 75:1, 30:1, 7.5:1, 1.5:1, and 0.75:1, respectively. PL spectra were measured, which is shown in Figure 2. It can be seen that all the CD emissions are excitation-independent, and the emission peaks are at around 580 nm, which also belongs to orange emission. The FWHM of the emission peaks are 35 (Figure 2a), 30 (Figure 2b), 32 (Figure 2c), 35 (Figure 2d), and 33 nm (Figure 2e), respectively, which are all less than 60 nm. When the molar ratio of phloroglucinol and rhodamine B is 30:1, the FWHM of the emission peaks of the OCDs is the narrowest (30 nm), which was measured ten times, and the standard deviation is 0.2989. Moreover, the PL spectrum of rhodamine B in ethanol was measured, the emission peak appears at 642 nm, and its FWHM is 51 nm (Figure 2f). It proved that the emission and FWHM of OCDs originate from the new products produced from phloroglucinol and rhodamine B, not the rhodamine B resource only. Moreover, the FWHM of the fluorescence spectra of OCDs is related to the number and type of their surface functional groups and the sp^2^ conjugation of the carbon core, etc. The fluorescence emission of OCDs mainly originated from different luminescent centers, such as a rhodamine-B-derived structure and other functional groups on the surface. Therefore, the FWHM of OCDs is mainly correlated with their surface structure. Since rhodamine B has a small FWHM, the broadening of the emission spectrum of OCDs mainly originates from other luminescent centers on the surface of OCDs. It can be inferred that when the raw material molar ratio is 30:1, the fluorescence emission of OCDs with the highest content of fluorescence emission originated from the luminescent centers on the rhodamine-B-derived structure, which means that they have narrowest emission spectrum. OCDs obtained under another molar ratio have a large number of luminescent centers with non-rhodamine-B-derived structures, which have a broad emission spectrum.

It can be concluded that OCDs prepared in ethanol with the optimized reactant molar ratio of 30:1 have the narrowest FWHM of 30 nm and a QY of 27.31%, which shows better performance than the OCDs prepared in water. It can be illustrated in two ways: First, phloroglucinol has better solubility in ethanol, which makes it fully dehydrated and carbonized during the reaction process. Second, ethanol contains a large amount of –OH, and it can promote the generation of oxygen-containing functional groups on OCDs in the reaction process, which is conducive to narrowing the FWHM of OCDs [16]. Subsequently, the optimized obtained OCDs were used to conduct the following experiments.

### 2.2. Structure and Composition of OCDs 

**Figure 3:**
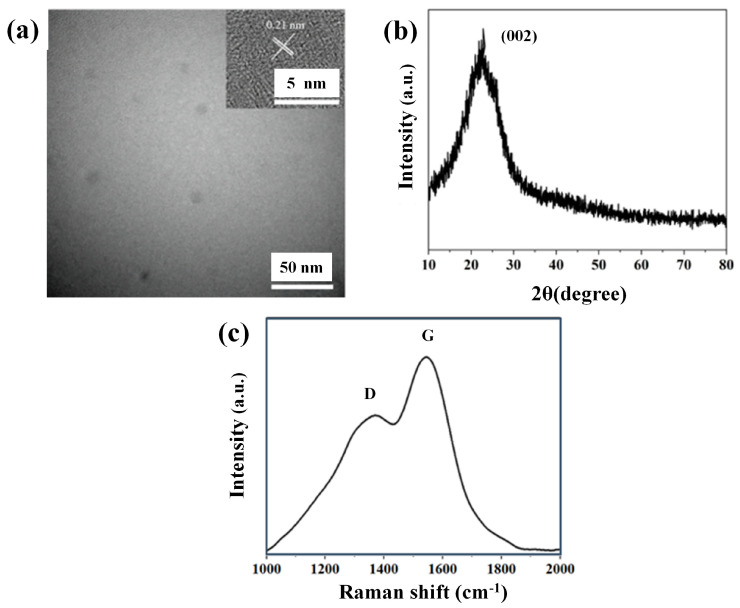
(**a**) TEM image (inset is HRTEM), (**b**) XRD pattern, and (**c**) Raman spectrum of OCDs.

The micromorphology and structure of OCDs were characterized by transmission electron microscopy (TEM) and high-resolution transmission electron microscopy (HRTEM) (Figure 3a). It shows that OCDs are spherical particles with a size of about 10 nm. It can be seen clearly from the HRTEM image that the lattice space is 0.21 nm, which is attributed to the (100) crystal plane of graphite, suggesting that the OCDs possess microstructural characteristics similar to that of graphite. To further analyze the crystalline phase composition of the OCDs, the X-ray diffraction (XRD) pattern was measured (Figure 3b). A distinct diffraction peak at 22.29° is observed, which is not as sharp as graphite’s characteristic peak near 26.5°, but it is not as flat as that of completely disordered carbon material (such as activated carbon), indicating that the OCDs still have a certain degree of graphitization. The crystal spacing was calculated to be 0.398 nm using Bragg’s equation, which may be due to the presence of surface groups or luminescent centers in OCDs expanding their crystal spacing. In addition, Raman spectrum was carried out to further verify the crystalline structure of OCDs, as shown in Figure 3c. Two prominent peaks at 1360 and 1620 cm^−1^ appear in the Raman spectrum, which correspond to the D and G bands, respectively. The D band represents the defects in the carbon nanomaterials, and the G band represents the sp^2^ hybridization of the carbon nanomaterials. The ratio of I_G_/I_D_ represents the graphitization degree of the OCDs, and the larger the ratio is, the higher the graphitization degree of the OCDs is. The I_G_/I_D_ ratio of OCDs is 1.41, proving that OCDs have a graphitized structure and good crystallinity, which is also an important factor for obtaining narrower FWHM of the emission peak of CDs [7].

**Figure 4:**
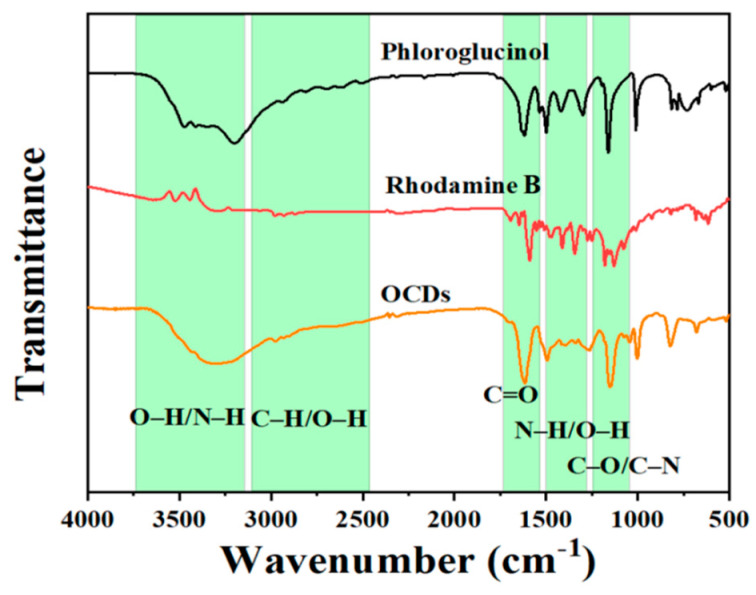
FT-IR spectra of phloroglucinol, rhodamine B, and OCDs.

The surface composition of OCDs was analyzed by Fourier transform infrared spectrometer (FT-IR) characterization. The FT-IR spectra of phloroglucinol, rhodamine B, and OCDs are shown in Figure 4. It can be seen that the surface of OCDs has a relatively abundant oxygen-containing functional groups. The absorption band at 3330 cm^−1^ is attributed to the O–H and N–H antisymmetric stretching vibration peak [17], and the absorption bands at 1610, 1396, and 1205 cm^−1^ are attributed to the C=O and C–N stretching vibration peak and the asymmetric stretching vibration of the aromatic ether C–O–C [18], respectively, indicating the generation of N- and O-conjugated aromatic rings in OCDs [19]. Compared with rhodamine B and phloroglucinol, the OCDs have a large amount of C=O. Moreover, compared with rhodamine B with narrow emission, the –OH and C–O–C contents of OCDs are higher, which are beneficial for obtaining OCDs with narrow FWHM [20].

**Figure 5:**
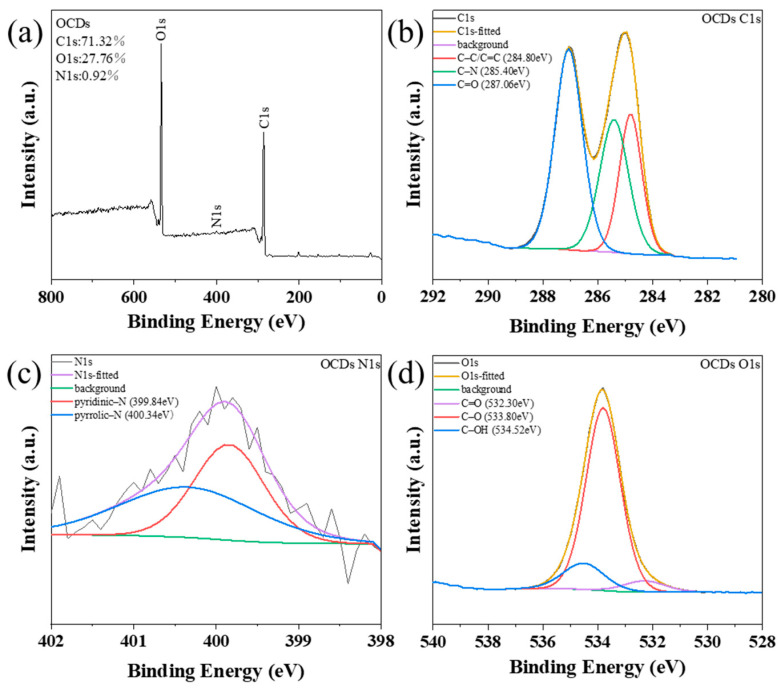
(**a**) XPS spectrum and (**b**–**d**) high-resolution XPS C1s, N1s, and O1s spectra of OCDs.

To further confirm the surface elemental composition of OCDs, X-ray photoelectron spectroscopy (XPS) spectra were analyzed (as shown in Figure 5). Three typical peaks can be observed from their full spectrum (Figure 5a): the C1s (285.08 eV), N1s (400.08 eV), and O1s (533.08 eV). The relative contents of C, O, and N elements are 71.32%, 27.76%, and 0.92%, respectively. Further, the high-resolution C1s spectrum shows that there are C–C/C=C (284.80 eV), C–N (285.66 eV), and C=O (287.32 eV) in OCDs (Figure 5b), in which the proportion of C–C/C=C and C=O is more than 70%, indicating that OCDs have large sp^2^-conjugated carbon structures. In the high-resolution N1s (Figure 5c), there are pyridine nitrogen (399.84 eV) and pyrrole nitrogen (400.34 eV) in OCDs, suggesting that the N element in rhodamine B dopes into OCDs. In the high-resolution O1s spectrum, there are C=O (532.30 eV), C–O (533.80 eV), and C–OH (534.52 eV) in OCDs, indicating the presence of –COOH and C–O–C in OCDs (Figure 5d), which is consistent with the characterization of the FT-IR spectra results.

### 2.3. Optical Properties of OCDs

**Figure 6:**
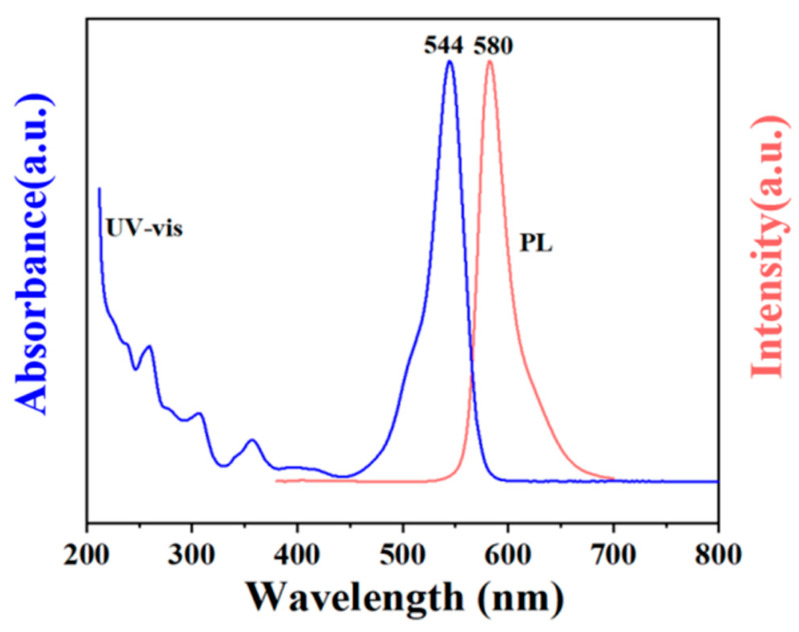
UV-vis and PL spectrum of OCDs.

To investigate the optical properties of OCDs, ultraviolet visible (UV-vis) and PL were characterized, as shown in Figure 6. The absorption peaks at 200 and 320 nm mainly originated from π-π* transitions, which was derived from phloroglucinol (Figure S1a) and some carbonized structure produced in the synthesis of OCDs. In addition, the absorption peaks at 350 nm attributable to the n-π* transitions of C=O/C=N and the absorption peaks at 450 nm and more than 450 nm originated from large sp^2^ conjugation [21,22], which is derived from rhodamine B (Figure S1b) and the conjugated carbon core. The fluorescence characteristics of OCDs are related to the sp^2^-conjugated structure and surface functional groups. According to Figure 6, the Stokes shift of OCDs is only 36 nm, and such a shift can help OCDs to produce high-quality solid-state lighting devices [13].

**Figure 7:**
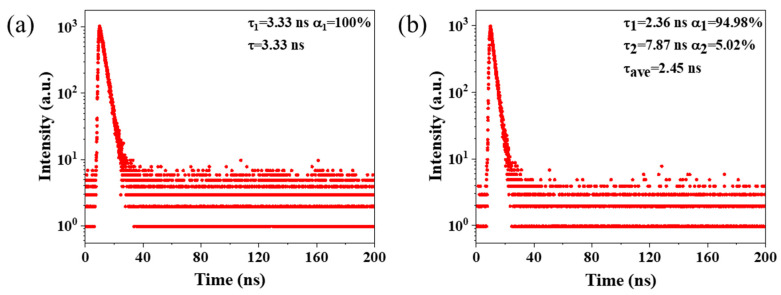
Fluorescence decay of (**a**) rhodamine B and (**b**) OCDs.

To investigate the source of the luminescence of OCDs, the fluorescence lifetimes of rhodamine B and OCDs were measured, as shown in Figure 7. The results indicate that rhodamine B exhibits a monoexponentially decay with a lifetime of 3.33 ns (Figure 7a), and OCDs show a biexponential decay with a fluorescence lifetime of 2.45 ns (Figure 7b). As shown in Figure 7b, τ_1_ has a lifetime of 2.36 ns, suggesting τ_1_ may derive from a rhodamine-B-related structure on the OCD surface, which can also be confirmed by the excitation spectrum of the OCDs and rhodamine B (Figure S2). That is, OCDs have almost the same excitation site of rhodamine B. The presence of other functional groups, such as hydroxyl and carboxyl groups, and organic molecules on the surface of OCDs can interact with rhodamine B and play a passivating effect on them, which in turn accelerates the radiative transition and reduces the lifetime. As a result, the fluorescence lifetime of OCDs originating from a rhodamine-B-related structure is a little smaller than that of rhodamine B in ethanol solution. At the same time, τ_2_ has a lifetime of 7.87 ns, which originates from other luminescent centers and defect states on the surface of OCDs. These results indicate that the emission of OCDs is mainly caused by its surface-defective state [23].

### 2.4. Optical Properties of OCD/PVA Films

**Figure 8:**
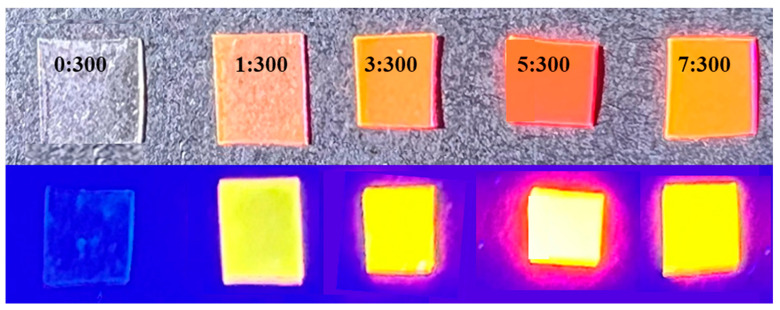
The photograph of OCD/PVA films in daylight (**up**) and under UV light (**down**).

Film-forming agents, such as PVA, can disperse CDs uniformly to inhibit the ACQ effect, thus achieving solid-state luminescence [5]. Therefore, a different concentration of OCDs were dispersed in PVA to prepare the OCD/PVA films with mass ratios of OCDs and PVA (0:300, 1:300, 3:300, 5:300, 7:300). Figure 8 shows the photographs of OCD/PVA films under daylight and UV lamp irradiation, respectively. It can be seen that the addition of OCDs makes the color of the films change from yellow to orange with the increased concentration of OCDs under daylight. Moreover, the OCD/PVA film luminescence is brightest at 5:300 under UV light.

**Figure 9:**
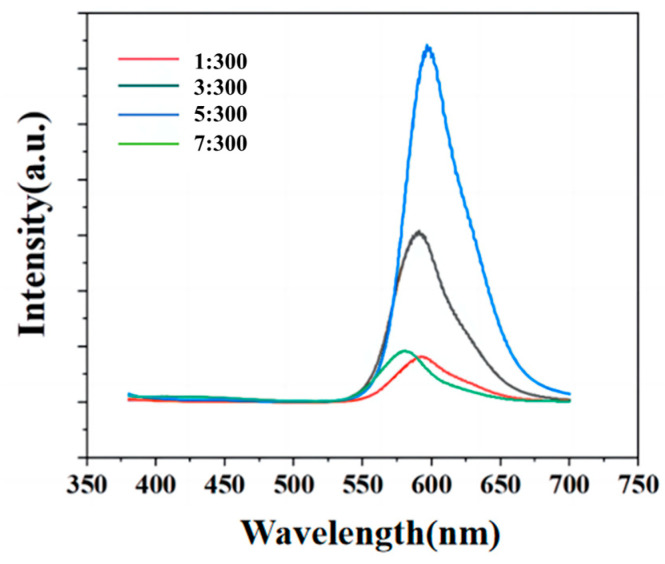
PL spectra of OCD/PVA films under different mass ratios (Ex: 385 nm).

The PL spectra of OCD/PVA films were further characterized (Figure 9). It can be seen that the luminescence intensity of OCD/PVA films first increases and then decreases with the increase in OCDs concentration in PVA. When the mass ratio of OCDs and PVA is 5:300, the OCD/PVA film shows the highest luminescence intensity. It can be attributed to the fact that when the concentration of OCDs is low (from 1:300 to 3:300), the OCDs can be well dispersed in PVA, and the luminescence intensity increases gradually. When the concentration of OCDs is high (from 5:300 to 7:300), the OCDs begin to aggregate and induce the ACQ effect, causing the decrease in the luminescence intensity of the OCD/PVA film. In addition, when the mass ratio of OCDs to PVA is 5:300, the emission peak FWHM of OCD/PVA film is the lowest (47 nm), and the QY is up to 84.74%, which has a great improvement compared with the QY of OCDs (27.31%). It confirms that PVA is a good film-forming agent to effectively inhibit the ACQ effect of OCDs and, thus, greatly enhance the luminescence intensity of the films. In the following experiments, the as-obtained OCD/PVA film with the mass ratio of 5:300 is characterized. Moreover, CDs synthesized using similar raw materials or solvents as this work are listed and compared by their optimal emission peak, FWHM, and QY in Table 1. The FWHM of the synthesized OCDs and their film is 47 nm (it was measured ten times, and the standard deviation is 0.2908), which is less than 60 nm and is a narrow bandwidth emission. As can be seen in Table 1, although the emission wavelength, FWHM, and solid-state QY of OCDs and their film are not the most outstanding, they still show superiority in CDs with long wavelength emission, narrow FWHM, and high solid-state QY simultaneously.

**Figure 10:**
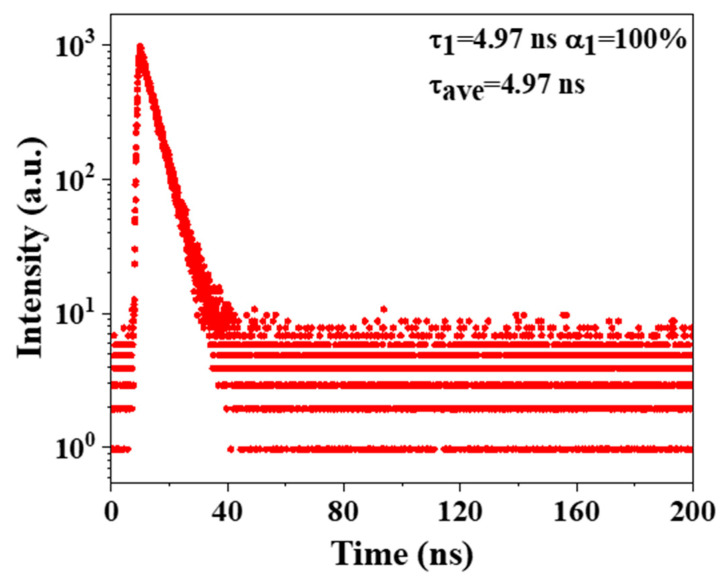
Fluorescence decay of OCD/PVA film.

In order to explore the luminescence source of OCD/PVA film, the fluorescence lifetime values were obtained, as shown in Figure 10. The fluorescence decay curve of this film shows monoexponential decay with a lifetime of 4.97 ns. The different lifetime values of OCDs and OCD/PVA film may originate from the surface structure change of OCDs, demonstrating that the surface state is the main luminescence center [28,29]. Additionally, the fluorescence lifetime of the film significantly increases, which illustrates that OCDs can disperse in PVA efficiently, thus preventing the fluorescence resonance energy transfer, which greatly prolongs the fluorescence lifetime of the film [30].

**Figure 11:**
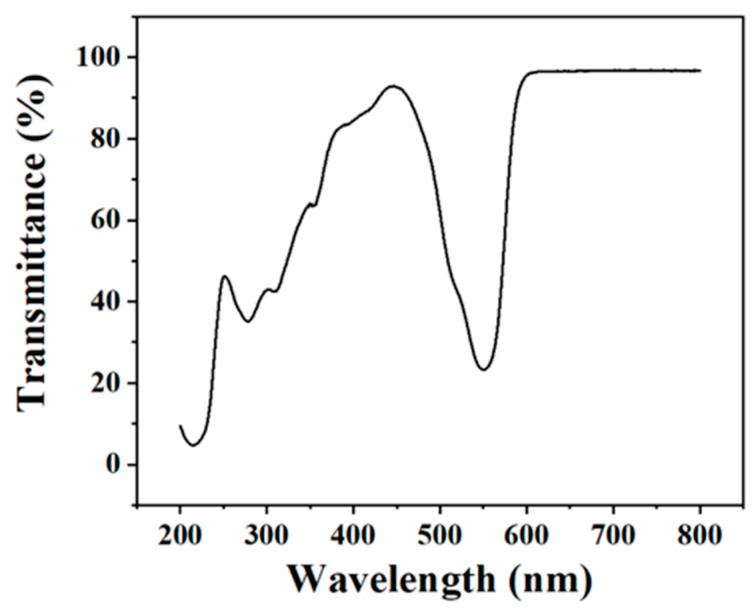
Light transmittance curve of OCD/PVA film.

The films with good transmittance can enhance the propagation of the light and reduce the optical loss, thus contributing to the solid-state lighting performance. Figure 11 shows the transmittance curve of the OCD/PVA film, and it can be seen that the film generally has low transmittance in the UV range, averaging below 40%, and most of the transmittance in the visible range is above 90%. The transmittance reaches a minimum value of 20% at 560 nm in the visible range, which is consistent with the luminescence peak position of the OCD/PVA film. In addition, the transmittance of the film at other positions in the visible range is 90%, which indicates that the OCD/PVA film has good light transmittance. This kind of film with low transmittance in UV light and high transmittance in visible light is very favorable for application in the lighting field, which not only reduces the loss of light, but also reduces the harm of UV light to the human body [31].

**Figure 12:**
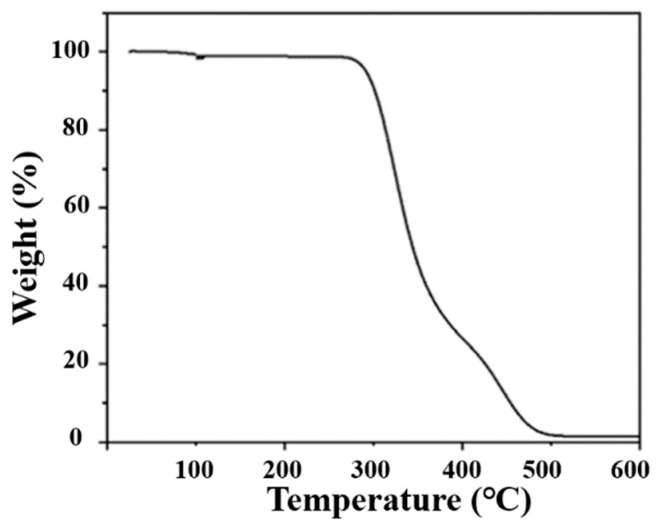
TG curve of OCD/PVA film.

Fluorescent film for the lighting field generally can work at between 20 and 150 °C [32]. Therefore, OCD/PVA film should have good thermal stability below 150 °C. In order to characterize the thermal stability of OCD/PVA film, thermogravimetric (TG) tests were performed. Figure 12 shows the TG curve of the OCD/PVA film. It can be seen that the film has almost no weight loss before 300 °C and about 30% weight loss at 400 °C, which is attributed to the decomposition of the PVA matrix in the film. The outcome indicates that the OCD/PVA film has excellent thermal stability.

**Figure 13:**
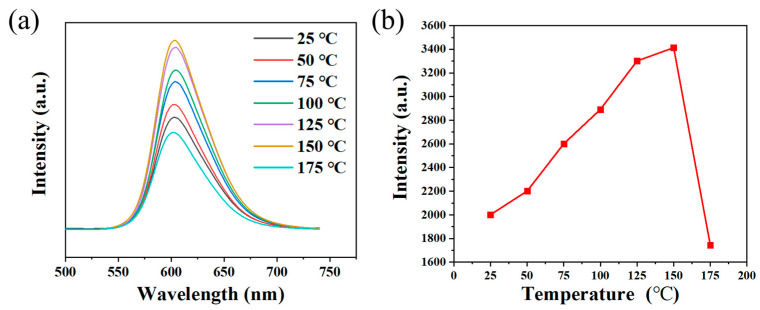
(**a**) PL spectra and (**b**) curve of fluorescence intensity change of OCD/PVA film under different temperatures.

Additionally, the fluorescence intensity of the film at different temperatures is also important for stable lighting devices [33]. Therefore, the fluorescence intensities of OCD/PVA film at different temperatures were tested. Figure 13 shows the PL spectra of OCD/PVA film at different temperatures under the same excitation and the variation curves of their fluorescence intensities. It can be seen that the fluorescence intensity of film gradually increases from room temperature (25 °C) to 150 °C, reaches the top value at 150 °C, and then decreases sharply at 175 °C. It indicates that the film has high fluorescence stability and is suitable for the working environment from 25 to 150 °C. This may be because the thermal burst phenomenon causes some excited-state electrons to undergo non-radiative transitions at 175 °C, which return to the ground state without emitting photons, thereby leading to a reduction in fluorescence intensity [34]. In conclusion, the OCD/PVA film has excellent thermal stability and is suitable for the operating temperature of lighting devices.

### 2.5. Formation and Luminescence Mechanism of OCDs and Their Films

**Figure 14:**
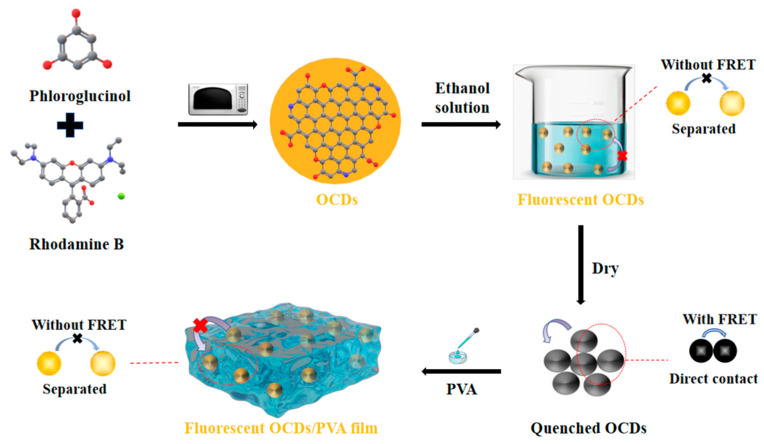
Schematic diagram of the formation process of OCDs and OCD/PVA films; mechanism diagram of solid-state luminescence OCDs, quenched OCDs, and fluorescent OCD/PVA film.

Combined with the above characterization results of OCDs and their fluorescent films, their formation and luminescence mechanism are further explored. As depicted in Figure 14, the highly symmetric phloroglucinol was used as the carbon source to prepare OCDs, which provides OCDs with a big conjugate size and high symmetry. It is beneficial for obtaining CDs with long-wavelength emission and narrow FWHM. The luminescence of OCDs originates from surface-state luminescence; wherein, the surface is enriched with diverse functional groups, including –OH from phloroglucinol and –COOH and C–O–C from rhodamine B. These abundant oxygen-containing functional groups facilitate the formation of OCDs with a narrow FWHM. After compositing with PVA, the OCDs can be uniformly dispersed in the PVA matrix to form OCD/PVA film. The strong interactions between the OCDs and the PVA chains are facilitated through hydrogen bonding. OCDs embedded into the PVA matrix can form an ordered mesh structure, and the vibration of the PVA chains is restrained owing to the hydrogen bonding effect, thus enhancing the thermal stability of the OCD/PVA film [35,36,37].

The solid-state luminescence mechanism of OCD/PVA film is also shown in Figure 14. When OCDs are dissolved in ethanol solution, the OCD particles are well dispersed in ethanol, and there is no direct contact among different OCDs. Therefore, the ethanol solution of OCDs show fluorescence emission. However, when OCD powders are obtained by drying, the OCDs tightly cluster together with a distance less than the Foster distance (1–10 nm) [38], which is accompanied by the occurrence of fluorescence resonance energy transfer (FRET), leading to the ACQ effect. In contrast, in OCD/PVA films, OCDs can be well disperse in PVA with a higher dispersion than in solution, resulting in a suitable distance between OCDs, which is larger than the Foster distance to avoid the occurrence of FRET. Therefore, the ACQ effect of the OCDs is consequently suppressed, and the OCD/PVA films with higher QY are obtained. This result provides a theoretical basis for their application in the solid-state fluorescence lighting field.

## 3. Experimental

### 3.1. Materials

Phloroglucinol and rhodamine B were purchased from Shanghai McLean Biochemical Technology Co., Ltd., Shanghai, China. Anhydrous ethanol was purchased from Shanghai Aladdin Biochemical Technology Co., Ltd., Shanghai, China. PVA was purchased from Sinopharm Chemical Reagent Co., Ltd., Shanghai, China Deionized water was produced in our laboratory. All the chemical reagents in this experiment are analytically pure and were not further processed.

### 3.2. Characterization

TEM and HRTEM image were recorded on a JEM-2010, JEOL, Tokyo, Japan. XRD patterns were obtained in a D/MAX 2500, Rigaku, Japan. Raman spectra were measured with excitation of 325 nm on a LabRAM HR Evolution Raman spectrometer, Horiba, Longjumeau, France. The FT-IR spectra in this paper were measured on a Tensor 27 spectrometer, Bruker, Bremen, Germany, by making dilutions of OCD power in solid KBr and with subsequent pressing to prepare disks of these materials, which are visually clear to characterize. XPS spectra were obtained on a Thermo Scientific ESCALAB 250Xi, Thermo fisher scientific, Waltham, MA, USA. UV-vis absorption spectra were recorded on a U-3900 UV-vis spectrophotometer, Hitachi, Japan. The fluorescence decay and lifetime were recorded on an FLS1000, Edinburgh Instruments, Livingston, UK. The PL emission spectra were acquired by an FS5 fluorescence spectrometer, Edinburgh, UK, and the QY values of OCDs ethanol solution and OCD/PVA film were measured using the integrating sphere module with a Xenon lamp as excitation source (Ex: 385 nm). The TG analysis was conducted by a DTG-60AH, Shimadzu, Japan, in a nitrogen atmosphere with 4.2476 mg of OCD/PVA film, and the weight loading of OCDs in the film is 1.63%. The transmittance results of films were collected on UV-VIS-NIR, PElambda750, PerkinElmer, Waltham, MA, USA.

### 3.3. Synthesis of OCDs with Narrow FWHM

OCDs were synthesized by microwave method using phloroglucinol as precursor, rhodamine B as dopant, and water or ethanol as reaction solvent. An amount of 0.63 g of phloroglucinol and 0.08 g of rhodamine B (molar ratio 30:1) were added into 20 mL of solvents in a 100 mL beaker to obtain the uniform mixture by ultrasonication for 20 min. Further, the beaker was put into a microwave oven and heated for 10 min at 640 W. After the reaction, once it was cooled to room temperature, 20 mL of solvents were added to the beaker to dissolve the product with ultrasonication for 5 min. Subsequently, the solution was filtered by a micropore filtration membrane of 0.22 μm to remove byproducts. Finally, the solution was purified by a dialysis bag with a molecular weight cut-off of 500 D for 2 d in solvent, and then, the solvent was evaporated to obtain OCD powder. During this experiment, the effects of modulating the molar ratio of phloroglucinol and rhodamine B (75:1, 30:1, 7.5:1, 1.5:1, and 0.75:1) and reaction solvent type (water and ethanol) on the FWHM of OCDs were examined, separately.

### 3.4. Preparation of OCD Fluorescent Films

OCD fluorescent films were prepared by the solution casting method, in which OCD/PVA films with different mass ratios of OCDs and PVA (0:300, 1:300, 3:300, 5:300, and 7:300) were prepared. First, 0, 2, 6, 10, and 14 mg of OCD powder were placed in five 5 mL beakers, separately, and then, 3 mL of deionized water and 0.6 g of pure PVA particles (MW: 85,000–124,000) were added. Second, it was placed on a hot table and stirred at 80 °C for 30 min to obtain the OCD/PVA composite solution. Third, the solution was dried in a vacuum oven at 60 °C for 6 h to obtain OCD/PVA composite films with different mass ratios. The films were cut into suitable sizes and packed into sealed bags for subsequent characterization and performance testing.

## 4. Conclusions

In this paper, OCDs were synthesized using the microwave method using phloroglucinol and rhodamine B. The optimal reaction parameters were found by investigating the effects of raw material molar ratios and reaction solvents on the FWHM of OCDs, and the morphology, structure, and optical properties of OCDs were characterized. The results showed that the spherical-like OCDs were obtained under the optimal parameters (reactant molar ratio of 30:1, reaction solvent of ethanol, reaction time of 10 min, reaction power of 640 W). The optimized OCDs have good dispersion, optimal emission wavelength at about 580 nm, an FWHM of 30 nm, and a QY of 27.31%. The source of the luminescence of the OCDs was mainly dependent on the surface state with a fluorescence lifetime of 2.45 ns. In addition, in order to realize the solid-state luminescence of OCDs, OCD/PVA films were fabricated using the film-forming agent PVA to obtain homogeneous OCD/PVA fluorescent thin films with good transmittance and thermal stability. The optimized OCD/PVA film has an FWHM of 47 nm, a fluorescence lifetime of about 4.97 ns, and a QY of 84.74%. The OCDs and their films prepared in this work will show great potential for applications in the solid-state lighting field.

## Figures and Tables

**Table 1:** Optimal emission peak, FWHM, and QY of CDs and their films.

Precursor	Reaction Solvents	Emission (nm)	FWHM (nm)	Solid-State QY (%)	Refs
Phloroglucinol	1,2-Pentanediol	474	33	/	[10]
		494	35	/	
Resorcinol	Ethanol	520	31	/	[11]
		610	33	/	
*o*-Phenylenediamine	Acetic acid, Deionized water	308	24	20.20	[12]
*p*-Phenylenediamine	Ethanol	600	100	41.72	[24]
Phloroglucinol	N, N-dimethylformamide	550	38	17	[25]
*o*-Phenylenediamine	Chloroform	595	31	34.8	[26]
*m*-Phenylenediamine	Ethanol	521	41	80	[27]
Phloroglucinol Rhodamine B	Ethanol	580	47	84.74	This work

## Data Availability

All the data generated by this research are included in the article.

## Supplementary Materials

The following supporting information can be downloaded at: https://www.mdpi.com/article/10.3390/molecules29204787/s1, Figure S1: UV-vis spectrum of (a) phloroglucinol and (b) rhodamine B; Figure S2: Excitation spectrum of (a) OCDs and (b) rhodamine B.
