# Supplementary material for: Carbon Dots and Their Films with Narrow Full Width at Half Maximum Orange Emission
Source: Molecules. 2024 Oct 10;29(20):4787. doi: 10.3390/molecules29204787 (PMC11510958; doi:10.3390/molecules29204787)
Supplement: Supplementary file 1 [file molecules-29-04787-s001.zip › molecules-3233982-supplementary.pdf]

molecules

MDPI

Supporting information

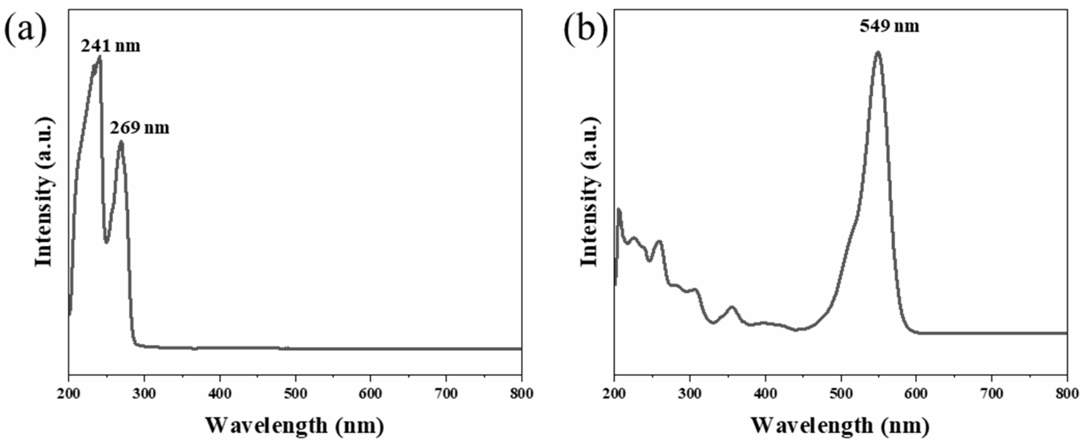

Figure S1. UV-vis spectrum of (a) phloroglucinol and (b) rhodamine B

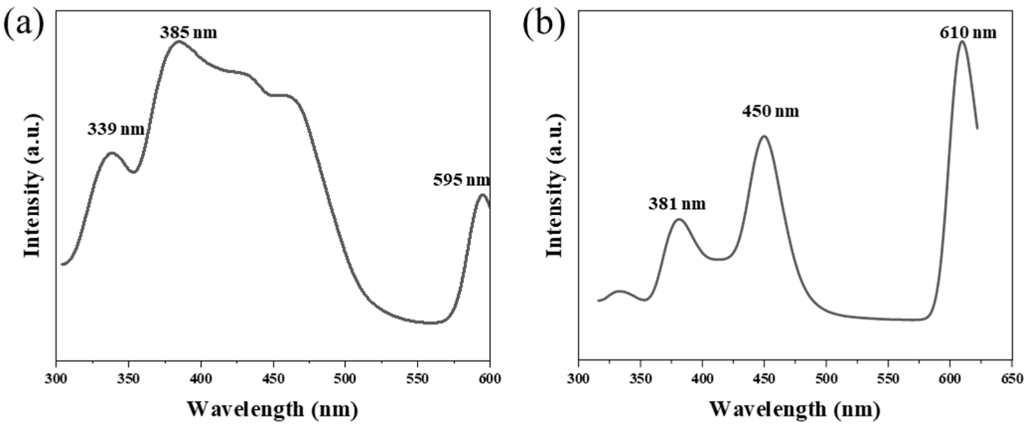

Figure S2. Excitation spectrum of (a) OCDs and (b) rhodamine B
